# Electronic Origin of α″ to β Phase Transformation in Ti-Nb-Based Thin Films upon Hf Microalloying

**DOI:** 10.3390/ma13061288

**Published:** 2020-03-12

**Authors:** José Julio Gutiérrez Moreno, Nikolaos T. Panagiotopoulos, Georgios A. Evangelakis, Christina E. Lekka

**Affiliations:** 1 Department of Materials Science and Engineering, University of Ioannina, 45110 Ioannina, Greece; 2 Department of Physics, University of Ioannina, 45110 Ioannina, Greece

**Keywords:** Ti alloys, β-phase, density functional theory, thin film, magnetron sputtering, X-ray diffraction, biocompatible materials

## Abstract

We present results on thin Ti-Nb-based films containing Hf at various concentrations grown by magnetron sputtering. The films exhibit α” patterns at Hf concentrations up to 11 at.%, while at 16 at.% Hf, the β-phase emerges as a stable structure. These findings were consolidated by ab initio calculations, according to which the α”–β transformation is manifested in the calculation of the electronic band energies for Hf contents between 11 and 18 at.%. It turns out that the β-phase transition originates from the Hf 5d contributions at the Fermi level and the Hf 6s hybridizations at low energies in the electronic density of states. Bonding–anti-bonding first neighbor features existing in the shifted plane destabilize the α″-phase, especially at high Hf concentrations, while the covalent-like features in the first neighborhood stabilize the corresponding plane of the β-phase. Thin films measurements and bulk total energy calculations agree that the lattice constants of both α″ and β phases increase upon Hf substitution. These results are important for the understanding of β-Ti-based alloys formation mechanisms and can be used for the design of suitable biocompatible materials.

## 1. Introduction

Ti-based alloys with non-toxic additions are potential candidates for biomedical applications due to their biocompatibility and low-rigidity features [1,2,3]. The desired low Young’s modulus is related to the stabilization of the β-Ti phase that is promoted by the presence of β-stabilizer elements such as Nb [2,3,4], Mo [5,6] or Cr [7,8,9]. In particular, Ti-Nb alloys with low Nb content exhibit several hexagonal or tetragonal phases (such as α, ω or α”) [10,11,12,13,14], while Ti-Nb_x_ (22 < x< 95 at.%) thin films exhibit mainly the β-cubic phase [15,16]. Implants’ surfaces play an extremely important role in the response of the biological environment to artificial medical devices [17,18]. In titanium implants, typical manufacturing steps usually lead to an oxidized and contaminated surface layer. These surfaces are often stressed and plastically deformed non-uniformly and are rather poorly defined [1,19]. 

Apart from Nb, Hf is another non-toxic element that is fully miscible to Ti forming α + β solid solutions, while its chemical similarities with Ti and its excellent corrosion resistance render the Ti-Hf system an interesting passive film for biomedical applications [20,21]. The oxidation of the Ti-Hf films depends on the composition, while the X-ray diffraction (XRD) measurements exhibit mainly the α′-phase for these films without intermediate compound structures [22]. Interestingly, small amounts of Hf can slightly affect the Ti-Hf’s dynamic Young’s moduli, while they increase the tensile strength of the system [23]. 

Ti-Nb-Hf ternary alloys have a strong potential to combine the biocompatibility of these three elements. Especially in the thin film form, these Ti-Nb-Hf alloys could be of valuable use as coatings. However, to our knowledge, reports focusing on the stabilization of the β-phase of Ti-Nb-Hf are rather scarce. Aiming to reveal the preferred phases and their electronic origin, we present a combined theoretical and experimental study on Ti-based systems with low Nb substitutions (around 17 at.%—where the α” phase emerges in the Ti-Nb alloys) and various Hf content (between 5.55 and 25.00 at.%). Thin Ti-based films were grown by magnetron sputtering (MS) and characterized by XRD measurements and energy dispersive X-ray spectroscopy (EDS). Linearly augmented plane wave density functional calculations DFT-LAPW TiNb_18.75_Hf_x_ (0 ≤ x ≤ 25 at.%) were used to reveal the preferred crystalline phases and their electronic origin. These results are adequate for understanding the β-Ti based alloy formation mechanisms and can be used for the design of suitable biocompatible materials.

## 2. Materials and Methods 

### 2.1. Experimental Details

The ternary Ti-Nb-Hf films were deposited on commercial, Czochralski-grown, n-type Si (001) provided by MaTecK Material-Technologie & Kristalle GmbH (Jülich, North Rhine-Westphalia, Germany). The Si wafers were cleaned in an ultrasound acetone and methanol bath, in sequence, followed by a dip in hydrofluoric acid (HF), rinse off by deionized water and then dried under high-pressure flow of high-purity nitrogen (purity 99.999%). The deposition was performed by dual-cathode confocal magnetron sputtering using high-purity Ti (purity 99.995%) and Nb (purity 99.8%) magnetron targets in a high vacuum chamber (base pressure of 4 × 10^−6^ mbar). The ternary Ti-Nb-Hf films of various Hf atomic contents were grown by fitting high-purity Hf (purity 99.99%) foils of different width on the Ti sputtering target, covering that way a different percentage of the sputtering ring. High-purity Ar (purity 99.999%) was used as sputtering gas, which was leaked in the deposition chamber achieving working pressure of 4 × 10^−2^ mbar. The applied power to the magnetron guns was fixed at 80W RF and 15W DC for the Ti-Hf and the Nb targets, respectively. The compositions of the grown samples were determined by energy-dispersive X-ray spectroscopy (EDS) in a JEOL JSM-5260 scanning electron microscope (JEOL Ltd, Akishima, Tokyo, Japan) coupled with an X-act Oxford Instruments X-ray analyzer, while grazing incidence XRD measurements were performed in a Bruker D8-Advance diffractometer (Bruker AXS GmbH, Karlsruhe, Baden-Württemberg, Germany) for evaluation of the crystal structure of the grown Ti-based films.

### 2.2. Computational Details

We performed the full-potential linearized augmented plane-wave method in the framework of Density Functional Theory (DFT) with the WIEN2k package [24]. The exchange-correlation functional within the generalized gradient approximation (GGA) in the form given by Perdew, Burke, and Ernzerhof (PBE96) was employed [25]. As a first step, the self-consistent iteration was performed for total energy and charge convergences of 10^−6^ Ry and 10^−2^ e^−^, respectively, for the optimization of the volume and lattice parameters, keeping the atomic position constant within the crystalline lattice. Afterwards, the ionic and electronic relaxations began with a force convergence criterion of 5·10^−3^ Ry/Bohr, retaining the total energy and the charge convergence limits. The cut-off parameter for convergence of the basis was set up to R_mt_K_max_ = 8, where R_mt_ is the smallest atomic sphere radius in the unit cell and K_max_ is the magnitude of the largest k-vector in the equation.

We used several unit cells to reproduce the α” and β phases, with respect to the Hf substitutions and sampling of the Brillouin zone. In line with the thin films’ composition, the Nb content was kept at around 17 at.% and we substituted Ti by Hf from 5.55 up to 25 at.%. For example, for the β-phase, we used up to 18 basis atoms along with 19 × 6 × 6 (160) k-point meshes, respectively. For the smallest Hf composition Ti_78_Nb_17_Hf_6_, while for the highest Hf composition (Ti_57_Nb_19_Hf_25_). we used 16 basis atoms along with 12 × 12 × 12 (196) k-point meshes.

The band energy *E_b_* was calculated using the expression [26]:

(1) $$E_{b}=\int_{-\infty }^{E_{F}}\varepsilon N\left(\varepsilon \right)d\varepsilon$$

where *N*(*ε*) is the canonical electronic density of states (EDOS) calculated separately for the total, *d*, *p* or *s*-electrons, while *E_F_* stands for the Fermi level (*E_F_*).

## 3. Results and Discussion

### 3.1. Structural Properties

#### 3.1.1. X-Ray Diffraction Patterns

**Figure 1:**
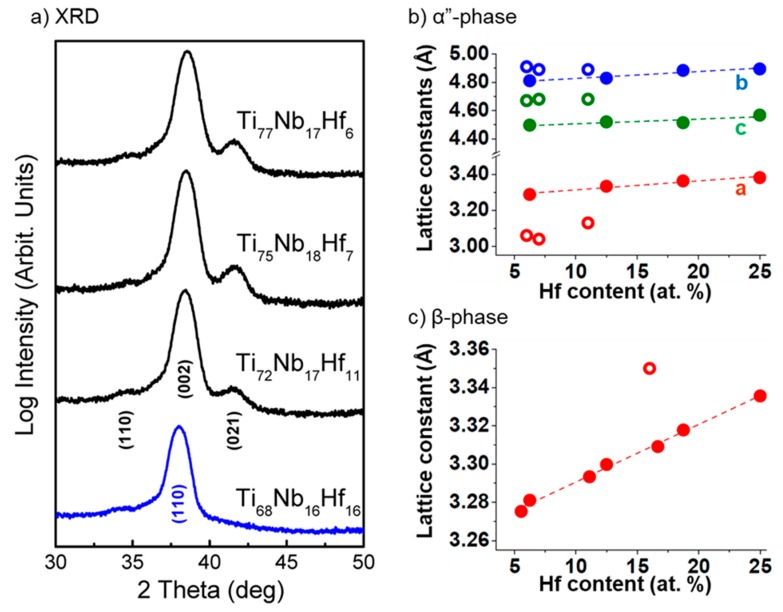
(**a**) Grazing incidence X-ray diffraction patterns of Ti-Nb-Hf films in α″-Ti (black lines) and β-Ti (blue line) crystal structure. Lattice constants versus Hf substitution for (**b**) α″ and (**c**) β phases. Filled and open circles correspond to ab initio and experimental data.

Figure 1 depicts the grazing incidence XRD patterns of the deposited Ti-based films. A textured growth is preferred for the direction of the [002] lattice planes in contrast to the bulk Ti-based alloys, for which the [110] peak was mainly observed [27,28,29]. For the study of the transformation of the α” to the β phase of Ti, the Nb content was kept below 25 at.% to prevent the growth of Nb stabilized β-Ti [30,31,32,33]. Additional elements of Nb and Hf in small amounts led to the growth of martensitic α” structure. Keeping the Nb content constant at 17 ± 1 at.%, and increasing the Hf content causes a small shift of the [002] peak for Hf content above 10 at.% and gradually adds to the deformation of the [021] lattice planes. For atomic Hf concentrations of 16 at.%, the observed (021) peak is completely deformed simultaneously with a strong peak shift of the (002) to larger lattice distance (lower angles of detection). This finding suggests that Hf substitutions of Ti atoms in the α″ structure induces strong deformation in the α″ lattice, eventually leading to a phase transformation towards the β-Ti. In Figure 1a, the Ti-16 at.% Nb-16 at.% Hf film (blue line) refers to β-Ti, since the peak observed corresponds (although shifted possibly due to the internal film stresses) to the [110] peak position of the β Ti-Nb structure. Indeed, tensile microstrain of 1.3% was calculated for the (110) peak position of the β Ti-Nb structure [30]. 

#### 3.1.2. DFT Calculations

From the equilibrium volume, we derived the crystals’ lattice constants. In Figure 1b, we present the ab initio results (filled circles) along with the available XRD data (open circles). The error of the experimental lattice parameters, as estimated from the determination of the XRD peak positions, is in the order of ±0.01 Å. All cases agree that upon Hf substitution, the individual α″ and β lattice constants increase almost linearly following Vegard’s law. Despite the difference in the lattice constant values between the DFT calculations and the experimental data, both models follow a similar trend and nearly identical slope.

**Figure 2:**
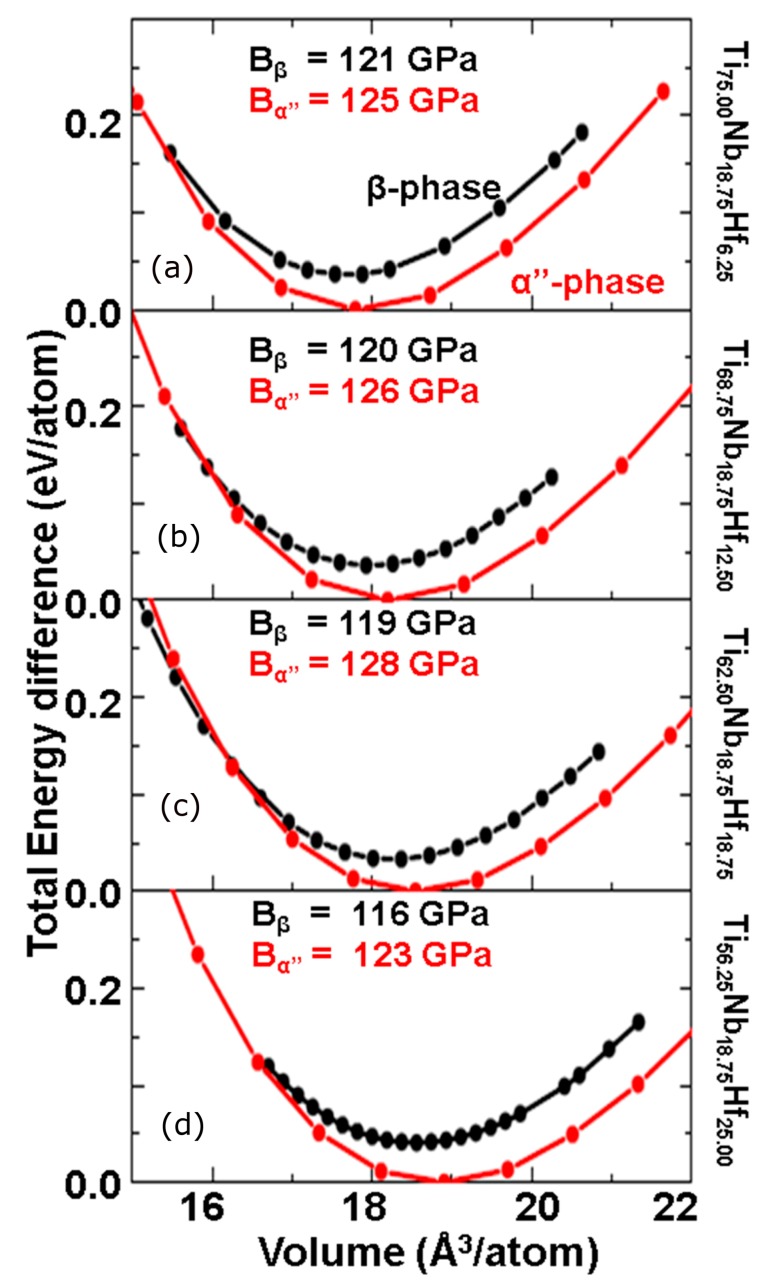
Total energy under hydrostatic pressure versus unit cell’s volume of (**a**) Ti-18.75 at.% Nb-6.25 at.% Hf, (**b**) Ti-18.75 at.% Nb-12.5 at.% Hf, (**c**) Ti-18.75 at.% Nb-18.75 at.% Hf and (**d**) Ti-18.75 at.% Nb-25 at.% Hf. Red and black points correspond to the α″ and β structures, respectively. The insets show the bulk moduli of the different lattices for all the compositions. The adjacent points in the graph are joined with straight lines.

The total energy for the α” and β structures subjected to hydrostatic pressure is presented in Figure 2. The α″ minimum energies have been shifted to zero and divided by the number of atoms for direct comparison. The α″-orthorhombic phase is energetically favored compared to the β-phase with energy differences around 0.05 eV/atom. Upon Hf substitution, the relative energy difference between the two phases decreases, suggesting the possible initiation of β-phase stabilizations in the bulk Ti-Nb-Hf system for Hf compositions up to 25 at.%. We observe a translation of the total energy minimum towards higher volumes in both phases, denoting a unit cell enlargement above 1 Å^3^/atom between the lowest (TiNb_18.75_Hf_6.25_) and the highest (TiNb_18.75_Hf_25.00_) Hf compositions.

The energy versus volume curves were fitted with the Birch–Murnaghan equation of state to determine the optimum lattice parameters (Figure 1b,c) and the ground state energy values. The bulk modulus (insets in Figure 2) was calculated from the first derivative of the fitting polynomial at its minimum. For the lowest Hf substitution, the β-phase bulk modulus (B_β_) is 121 GPa, with a decreasing trend reaching 116 GPa for 25 at.% Hf. For the orthorhombic phase, B_α__”_ slightly increases from 125 GPa for 6.25 at.% Hf to 128 GPa for 18.75 at.% Hf. B_α__′′_ gets its minimum value (123 GPa) for the highest Hf substitution (Ti-18.75 at.% Nb-25 at.% Hf). The B values for the α”-phase are higher than B_β_ for all the simulated compositions; however, considering the errors that can be carried in the estimation of the mechanical properties by ab initio calculations, these values can be considered as nearly constant [4].

**Figure 3:**
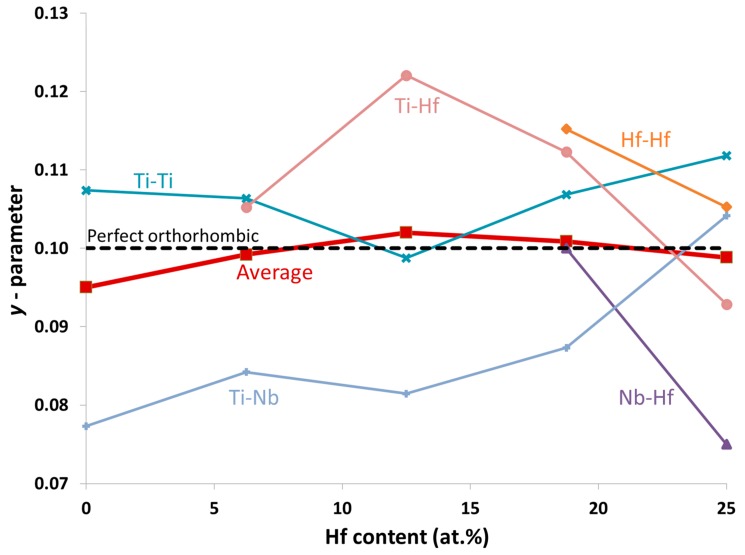
Orthorhombic y-parameter as a function of the Hf addition for Ti-18.75 at.% Nb-xHf. Above 11 at.% Hf, the y-parameter decreases denoting the α″ → β phase transition. The different lines correspond to each possible atomic pair.

The α″-orthorhombic structure can be considered as a transition between the hcp and bcc structures in the Ti-Nb alloy [34]. The orthorhombic structure in Ti-Nb (space group Cmcm, No. 63) can be constructed with four basis atoms at (0, 0, 0), (1/2, *y*, 1/2), (1/2, 1/2, 0) and (0, 1/2 *+* *y*, 1/2), where the y-parameter denotes the intermediate atomic shuffle within the lattice. For instance, the hcp structure is obtained when *y* = 1/6 and $b/a=\sqrt{3}$ while the bcc structure is obtained when *y* = 0 and $b/a=c/a=\sqrt{2}$ [35]. In Figure 3, the evolution of the y-parameter upon Hf addition is represented for the different first-neighbor pairs. The black dashed line depicts the (perfect) orthorhombic y-parameter (*y* = 0.10), experimentally suggested for Ti-Nb alloys [36]. 

The contribution from Ti-Nb pairs to the average y-parameter is more important at low Hf compositions, while in Hf-rich compounds, it becomes less significant due to the smaller weight of Ti-Nb into the average value. Ti-Nb pairs present values below 0.09 for Hf concentrations under 18.75 at.%, while at the highest Hf composition (25 at.%), *y* increases above 0.10. All Hf atoms, including pairs (Nb-Hf, Ti-Hf and Hf-Hf), contribute to the decrease of the total *y* values that is characteristic for the α” → β phase transition. Considering that all Hf containing pairs will be more abundant in Hf-rich alloys, this will lead to an overall decrease of y, which is related to the α” → β phase transition, in line with the present experimental results. Therefore, we conclude that at low Hf concentrations, the Ti-Nb pairs contribute to the transition from the α″ to β phase. The y-parameter decreases progressively as a result of the Hf substitution, with a main contribution of the Nb-Hf pairs. 

### 3.2. Electronic Properties

**Figure 4:**
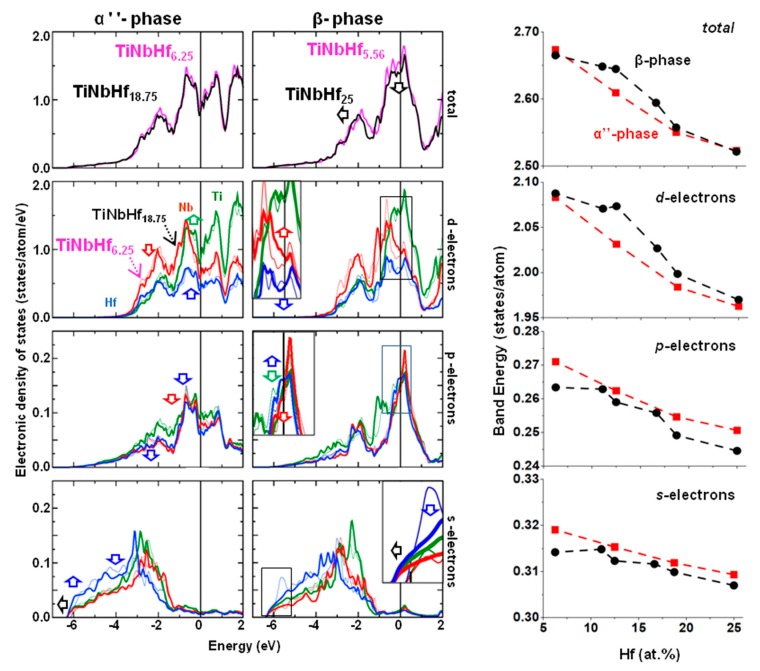
Total and partial electronic density of states (EDOS) of the α” (first column) and the β (second column) phases along with the electron band energy (third column). In the EDOS, magenta and black line stands for the lowest (6.26 at.%—thin line) and highest (25 at.%—thick line) Hf substitutions, while green, red, and blue stand for Ti, Nb, and Ti partial EDOS. The row sequence corresponds to the total, *d*, *p*, and *s* electron participation. The Fermi level is set to zero.

The electronic density of states (EDOS) and electronic band energy (*E_b_*) of the α”-phase (left panel) and β-phase (right panel) at low and high Hf substitutions are represented in Figure 4. The row sequence corresponds to the total and *d, p*, and *s* partial contributions, respectively. The most significant EDOS variations are denoted with arrows in the figure. The total and partial EDOS for all compositions and both crystalline structures are given as Supplementary Materials.

For the orthorhombic structure, the total EDOS is primarily influenced by the *d*-electrons, which contributes to an overall depletion of the states below *E_F_* at high Hf content (18.75 at.% Hf). Nb and Ti dominate the *d* partial EDOS, especially at energy states under −1.5 eV, below the *E_F_*. The α″-phase *d*-states with 6.25 at.% Hf are slightly enhanced at 0.5 eV below *E_F_*, whereas the opposite behavior is observed in the 18.75 at.% Hf composition further below *E_F_*. The sharp minimum of the α” EDOS can be related with the shape memory effect that characterizes the orthorhombic phase of Ti-Nb. The alterations induced in the partial *s* or *p*-states are minor compared to the *d*-states contribution. In the *p*–EDOS, all atoms contribute equally at energies near *E_F_*. The *p*-states between −1 eV and −3 eV are lowered upon Hf addition. The *s* states from Hf present their highest occupation under −3 eV. For the Hf-rich composition, the *s*-states at c.a. −6 eV are shifted by −0.25 eV compared to the low-Hf system. The small variations in the *s* and *p* partial EDOS are also observed in the band energy, where the decreasing slope is less pronounced than for the *d*-electrons. 

Turning on the β-phase, the Hf-rich total EDOS (black line) is depleted by approximately 0.3 states/eV/atom at *E_F_*, while the peak centered at around −2 eV is shifted towards lower energy states. These transitions are denoted by arrows in Figure 4. The described features are usually related to the enhancement of the stability of the β phase [26]. These variations will eventually result in a decrease of the electronic band energy. The Hf *d*-electrons are mainly responsible for the lowered electronic occupation at *E_F_* upon Hf substitution, as shown in the inset of the β-phase *d*-EDOS. On the other hand, Nb *d*-electrons are enhanced, while Ti atoms do not alter their contributions. Similarly to the α” phase, the *d*-electrons are mainly responsible for the main features of the total *E_b_*, while the *E_b_* variations between the lowest and highest Hf compositions are less than 7% for *s* and 3% for the *p*-electrons. The Ti and Nb atoms *p*-states are slightly depleted at *E_F_*, while Hf states are enhanced at this point. The *s*-electrons are responsible for the shift from −6 eV to −6.2 eV and the broadening of the peak centered at around −5.5 eV is also shifted towards lower energies. 

The estimation of the band energy (*E_b_*) provides valuable information about the structural preferences of the systems. Indeed, *E_b_* can designate the preference of a particular structure to another, by taking into account the shape and the electron-filling of the corresponding EDOS [26]. Major attention to the *d*-*E_b_* is usually justified by the fact that the most significant contributions in the total *E_b_* are due to the *d*-electrons. However, in this case, the *s*-*E_b_* and *p*-*E_b_* contributions are also important, especially in the lowest and the highest Hf contents (above 18.75 at.% Hf), as depicted in Figure 4. In fact, at 6.25 at.% Hf, there is a slight preference of the α″ phase compared to the β phase, while above 11 at.% Hf, the *E_b_* of the β-phase is clearly higher than the α″ phase, revealing the α″→ β transition, in line with our experimental observations. The *d*-*E_b_* is mainly responsible for this behavior, while the s-*E_b_* and p-*E_b_* suggest the α”-phase is always energetically favored. Nevertheless, above 18.75 at.% Hf, the β-*Ε_b_* significantly decreases, taking values slightly smaller or equivalent to α” at 25 at.% Hf, denoting instability of the β-phase and possible co-existence of the two phases. It seems, therefore, that the *E_b_* calculations may provide an alternative way to predict structural transitions of these experimentally very complex systems using the EDOS and *E_b_* calculations of β-perfect, ordered, small and simple unit cells.

**Figure 5:**
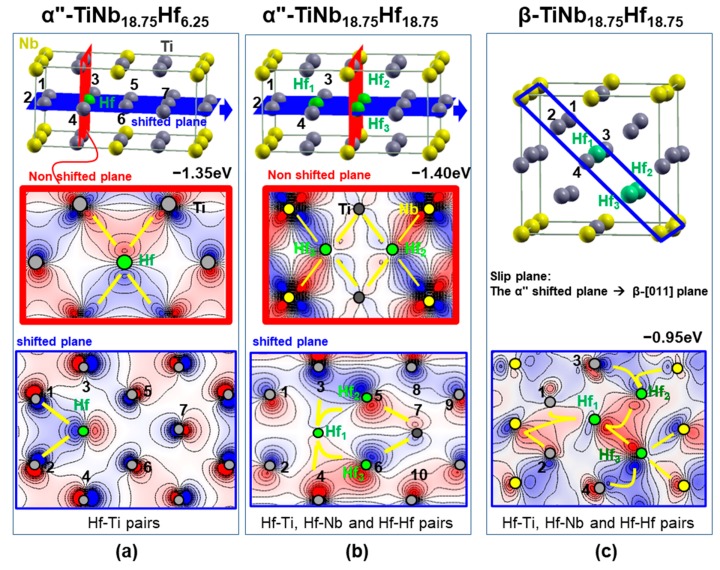
Schematic α″ and β phase structural representation and selective wave functions: (**a**) α″-TiNb_18.75_Hf_6.25_, (**b**) α″-TiNb_18.75_Hf_18.75_ and (**c**) β-TiNb_18.75_Hf_18.75_. Ti, Nb, and Hf atoms are presented with grey, yellow, and green, while the red and blue values stand for the negative and positive sign of the wave function (from −0.12 up to + 0.12 e/Å^3^).

In order to enlighten the role of electronic origin of the α” → β transition, we evaluated selective wave functions at the highest occupied EDOS peaks close to *E_F_*. Figure 5 shows two characteristic cases for αʹʹ-TiNb_18.75_Hf_6.25_ (with the favored Hf-Ti pairs) and αʹʹ-TiNb_18.75_Hf_18.75_ (with Hf-Hf, Hf-Ti, and Hf-Nb pairs) as well as the corresponding β-TiNb_18.75_Hf_18.75_ for comparison reasons. In the αʹʹ-TiNb_18.75_Hf_6.25_ crystal’s schematic representation, the characteristic αʹʹ shifted (blue plane) and a vertical non-shifted (red) plane at the Hf atom are depicted. Starting with the α″–TiNb_18.75_Hf_6.25_ non-shifted plane, the presence of strong directional covalent-like bonds between both Hf-Ti and Ti-Ti d-electrons’ pairs denotes the stability of this plane, Figure 5a. In contrast, in the shifted plane, although one *d* lobe of the Hf atom covalently-like shares its charge with the *d*-Ti lobes of atoms labeled 1 and 2, it exhibits anti-bonding characteristics with the 3, 4, 5, and 6 Ti atoms, revealing the instability of this plane. Similar features are also presented in the α″–TiNb_18.75_Hf_18.75_ case, Figure 5b. Directional covalent-like bonds are shown in the non-shifted plane between all first neighboring Hf-Ti and Hf-Nb pairs, while bonding (Hf1-Hf2, Hf1-Hf3, Hf1-Ti3, Hf1-Ti4, Hf2-Ti3, Hf2-Ti7, Hf3-Ti4, and Hf3-Ti7) and anti-bonding (Hf1-Ti1, Hf1-Ti2, as well as Ti7-Ti9, Ti7-Ti10) features are visible in the shifted plane between first- and second-neighbor pairs. Therefore, at high Hf concentration, the presence of Hf-Ti and Ti-Ti anti-bonding features in the shifted plane destabilizes the α″-phase towards a structure where possibly homogeneous bonding first-neighboring features will emerge. Indeed, Figure 5c depicts the corresponding disordered β-TiNb_18.75_Hf_18.75_, where directional bonding is manifested between the first-neighbor Hf-Hf, Hf-Ti, and Hf-Nb pairs, revealing the enhanced stability of the β-phase at this composition, against the α″-phase. The formation of bonding and anti-bonding first- and second-neighbor interactions is critical for the formation and stabilization of the β-phase in Ti-Nb alloys and directly related to the low rigidity of cubic crystalline structures [4]. The description of the wave functions is consistent with our previous studies on Ti-Nb-X ternary alloys with Sn [37] and In [38] additions, where the ternary substitutions lead to the formation of Ti-X local anti-bonding sites. At low X doping concentration, the bonding character of the binary Ti-Nb system is weakened, resulting in an elastic softening of the compound, while at higher X substitutions, the cubic Ti-Nb structure is destabilized [37,38]. Finally, these chemical features are expected to occur in the interfacial structure and successively change from anti-bonding pairs in the α″ shifted plane towards bonding pairs in the β phase, especially upon transformation.

## 4. Conclusions

We present a combination of theoretical and experimental data on the phase stability of α” or β phases of Ti-Nb-Hf. At low Hf compositions, the α”-phase is the preferred structure, while there is a critical composition range, between 11 and 16 at.% Hf according to the XRD measurements (between 11 and 18 at.% Hf from DFT), in which a transition from α″ to β phase was found. Both thin films measurements and bulk total energy calculations agree that the unit cell volume and the corresponding lattice constants increase upon Hf substitution. The overall decrease of the *y*-parameter suggests the progressive α” → β phase transition after the Hf addition. The physical insight of the β-phase manifestation and the interrelated destabilization of α″ is explained from the electronic structure of Ti-Nb-Hf alloys. We attributed these effects to the Hf hybridizations that decrease the total *d*-electron occupation at *E_F_* and reallocate the lowest energy *s*-electrons below −6eV, in conjunction with *p*-electron hybridizations occurring close to *E_F_*. The *E_b_* calculation yielded the α” → β phase transition above 11 at.% Hf, mainly due to the *d*-*E_b_* contribution, while above 18.75 at.% Hf, possible coexistence of the two phases is anticipated. In addition, the presence of bonding–anti-bonding features in the shifted plane destabilizes the α″-phase, especially at high Hf concentrations, while the experimentally observed preference towards the β-phase could also be explained due to covalent-like features at critical eigenvalues of the first neighborhood. At high Hf content, the enhanced number of Ti-Hf bonds may destabilize the structure, giving rise to the coexistence of α″ and β phases. These results could be of use for the design of β-Ti-based alloys with non-toxic additions, suitable for biomedical applications.

## Supplementary Materials

The following are available online at https://www.mdpi.com/1996-1944/13/6/1288/s1, Figure S1. Electronic density of states of α″-phase; Figure S2. Electronic density of states of β-phase.

## References

[1] Niinomi M. (2008). Mechanical biocompatibilities of titanium alloys for biomedical applications. J. Mech. Behav. Biomed. Mater..

[2] Niinomi M. (2002). Recent metallic materials for biomedical applications. Metall. Mater. Trans. A.

[3] Geetha M., Singh A., Asokamani R., Gogia A. (2009). Ti based biomaterials, the ultimate choice for orthopaedic implants—A review. Prog. Mater. Sci..

[4] Gutiérrez Moreno J.J., Papageorgiou D.G., Evangelakis G.A., Lekka C.E. (2018). An ab initio study of the structural and mechanical alterations of Ti-Nb alloys. J. Appl. Phys..

[5] Ho W.F., Ju C.P., Chern Lin J.H. (1999). Structure and properties of cast binary Ti–Mo alloys. Biomaterials.

[6] Oliveira N.T.C., Aleixo G., Caram R., Guastaldi A.C. (2007). Development of Ti–Mo alloys for biomedical applications: Microstructure and electrochemical characterization. Mater. Sci. Eng. A.

[7] Brozek C., Sun F., Vermaut P., Millet Y., Lenain A., Embury D., Jacques P.J., Prima F. (2016). A β-titanium alloy with extra high strain-hardening rate: Design and mechanical properties. Scr. Mater..

[8] Hong S.H., Park S.W., Park C.H., Yeom J.-T., Kim K. (2020). Relationship between phase stability and mechanical properties on near/metastable β-type Ti–Cr-(Mn) cast alloys. J. Alloy. Compd..

[9] Hong S.H., Hwang Y.J., Park S.W., Park C.H., Yeom J.-T., Park J.M., Kim K. (2019). Low-cost beta titanium cast alloys with good tensile properties developed with addition of commercial material. J. Alloy. Compd..

[10] Moffat D., Larbalestier D. (1988). The compctition between martensite and omega in quenched Ti-Nb alloys. Metall. Trans. A.

[11] Moffat D., Larbalestier D. (1988). The compctition between the alpha and omega phases in aged Ti-Nb alloys. Metall. Mater. Trans. A.

[12] Moffat D., Kattner U. (1988). The stable and metastable Ti-Nb phase diagrams. Metall. Mater. Trans. A.

[13] Lekka C.E., Gutierrez Moreno J., Calin M. (2017). Electronic origin and structural instabilities of Ti-based alloys suitable for orthopaedic implants. J. Phys. Chem. Solids.

[14] Gutiérrez Moreno J., Bönisch M., Panagiotopoulos N., Calin M., Papageorgiou D., Gebert A., Eckert J., Evangelakis G., Lekka C.E. (2017). Ab-initio and experimental study of phase stability of Ti-Nb alloys. J. Alloy. Compd..

[15] Mardare A.I., Savan A., Ludwig A., Wieck A.D., Hassel A.W. (2009). High-throughput synthesis and characterization of anodic oxides on Nb–Ti alloys. Electrochim. Acta.

[16] Photiou D., Panagiotopoulos N.T., Koutsokeras L., Evangelakis G.A., Constantinides G. (2016). Microstructure and nanomechanical properties of magnetron sputtered Ti−Nb films. Surf. Coat. Technol..

[17] Liu X., Chu P.K., Ding C. (2004). Surface modification of titanium, titanium alloys, and related materials for biomedical applications. Mater. Sci. Eng. R Rep..

[18] Lorenzetti M., Pellicer E., Sort J., Baró M., Kovač J., Novak S., Kobe S. (2014). Improvement to the corrosion resistance of Ti-based implants using hydrothermally synthesized nanostructured anatase coatings. Materials.

[19] Niinomi M. (1998). Mechanical properties of biomedical titanium alloys. Mater. Sci. Eng. A.

[20] Massalski T., Subramanian P., Okamoto H., Kacprzak L. (1990). Binary Alloy Phase Diagrams.

[21] Cai Z., Koike M., Sato H., Brezner M., Guo Q., Komatsu M., Okuno O., Okabe T. (2005). Electrochemical characterization of cast Ti–Hf binary alloys. Acta Biomater..

[22] Mardare A.I., Ludwig A., Savan A., Wieck A.D., Hassel A.W. (2009). High-throughput study of the anodic oxidation of Hf–Ti thin films. Electrochim. Acta.

[23] Zhou Y.-L., Niinomi M., Akahori T. (2008). Changes in mechanical properties of Ti alloys in relation to alloying additions of Ta and Hf. Mater. Sci. Eng. A.

[24] Blaha P., Schwarz K., Madsen G., Kvasnicka D., Luitz J. (2001). WIEN2k: An Augmented Plane Wave Plus Local Orbitals Program for Calculating Crystal Properties.

[25] Perdew J.P., Burke K., Ernzerhof M. (1996). Generalized gradient approximation made simple. Phys. Rev. Lett..

[26] Söderlind P., Eriksson O., Wills J., Boring A. (1993). Theory of elastic constants of cubic transition metals and alloys. Phys. Rev. B.

[27] Chawla V., Jayaganthan R., Chawla A., Chandra R. (2009). Microstructural characterizations of magnetron sputtered Ti films on glass substrate. J. Mater. Process. Technol..

[28] Chawla V., Jayaganthan R., Chawla A., Chandra R. (2008). Morphological study of magnetron sputtered Ti thin films on silicon substrate. Mater. Chem. Phys..

[29] Song Y., Cho S., Jung C., Bae I., Boo J., Kim S. (2007). The structural and mechanical properties of Ti films fabricated by using RF magnetron sputtering. J. -Korean Phys. Soc..

[30] Guo Y., Georgarakis K., Yokoyama Y., Yavari A. (2013). On the mechanical properties of TiNb based alloys. J. Alloy. Compd..

[31] Banumathy S., Mandal R.K., Singh A.K. (2009). Structure of orthorhombic martensitic phase in binary Ti–Nb alloys. J. Appl. Phys..

[32] Tobe H., Kim H., Inamura T., Hosoda H., Nam T., Miyazaki S. (2013). Effect of Nb content on deformation behavior and shape memory properties of Ti–Nb alloys. J. Alloy. Compd..

[33] Bönisch M., Calin M., Waitz T., Panigrahi A., Zehetbauer M., Gebert A., Skrotzki W., Eckert J. (2016). Thermal stability and phase transformations of martensitic Ti–Nb alloys. Sci. Technol. Adv. Mater..

[34] Morniroli J., Gantois M. (1973). Investigation of the conditions for omega phase formation in Ti–Nb and Ti–Mo alloys. Mémoires Et Études Sci. De La Rev. De Métallurgie.

[35] Li C.-X., Luo H.-B., Hu Q.-M., Yang R., Yin F.-X., Umezawa O., Vitos L. (2013). Lattice parameters and relative stability of α ″phase in binary titanium alloys from first-principles calculations. Solid State Commun..

[36] Ahmed T., Rack H. (1996). Martensitic transformations in Ti-(16–26 at %) Nb alloys. J. Mater. Sci..

[37] Gutiérrez Moreno J.J., Guo Y., Georgarakis K., Yavari A.R., Evangelakis G.A., Lekka C.E. (2014). The role of Sn doping in the β-type Ti–25 at % Nb alloys: Experiment and ab initio calculations. J. Alloy. Compd..

[38] Calin M., Helth A., Gutierrez Moreno J.J., Bönisch M., Brackmann V., Giebeler L., Gemming T., Lekka C.E., Gebert A., Schnettler R. (2014). Elastic softening of β-type Ti–Nb alloys by indium (In) additions. J. Mech. Behav. Biomed. Mater..

