# Supplementary material for: Electronic Origin of α″ to β Phase Transformation in Ti-Nb-Based Thin Films upon Hf Microalloying
Source: Materials (Basel). 2020 Mar 12;13(6):1288. doi: 10.3390/ma13061288 (PMC7142957; doi:10.3390/ma13061288)
Supplement: Supplementary file 1 [file materials-13-01288-s001.pdf]

materials

MDPI

Supplementary Materials

Electronic Origin of α'' to β Phase Transformation in Ti-Nb-Based Thin Films upon Hf Microalloying

José Julio Gutiérrez Moreno [1,*,†], Nikolaos T. Panagiotopoulos [2,‡], Georgios A. Evangelakis [2] and Christina E. Lekka [1,*]

[1] Department of Materials Science and Engineering, University of Ioannina, Ioannina 45110, Greece
[2] Department of Physics, University of Ioannina, Ioannina 45110, Greece
[*] Correspondence: juliogutierrez@szu.edu.cn (J.J.G.M.); chlekka@cc.uoi.gr (C.E.L.)
[†] Current address: Institute for Advanced Study, Shenzhen University, Shenzhen 518060, China
[‡] Current address: University of Cambridge, Department of Materials Science & Metallurgy, Cambridge CB3 0FS, UK

Density of States of relaxed and unrelaxed Ti-Nb-Hf α'' and β-phase structures

The Electronic Density of States' (EDOS) is calculated for the energetically favoured atomic conformation and the equilibrium lattice of all compositions and structures. In Figures S1 and S2, we present the α'' and β Ti-18.75at.%Nb-xHf (x = 6.25, 12.50, 18.75 and 25.00 at.%) total and partial DOS. The row sequence corresponds to the total, *d*, *p* and *s* electron contributions, while the perfect structure and the structure after ionic relaxation EDOSs are respectively depicted by thin and bold traces.

In Figures S1 and S2 we observe that the α'' and β phases exhibit low electron occupation (pseudo-gap) at the E_F and higher occupation of states bellow E_F, especially at high Hf compositions. These features are characterized by a highly occupied local minima at E_F, which is visible in α'' and β Ti-18.75at.%Nb-(6.25 ≤ x ≤ 25) at.% Hf compositions, revealing the metastable character of these structures. From the partial EDOS, Nb *d*-electrons are distributed between -3.5eV and E_F with the presence of two main peaks at −1 eV and −2 eV. The *d*-Ti-Nb-Hf hybridizations are mainly responsible for the states around E_F and subsequently responsible of the stabilization or destabilization of the different structures. Nb and Ti dominate the *d*-EDOS, especially below −1.5eV, while in the *p*-EDOS all atoms contribute equally close to the Fermi level. The partial *p*-electrons contribution is about 10 times smaller compared to *d*-electrons, although it also contributes to the depletion of state at E_F. The *s* contribution to the total EDOS is also smaller compared to the *d*-electrons. Hf has its highest occupation at the lowest energies of the *s*-EDOS. The Ti-Nb-Hf hybridizations are also visible in the *s*-electrons, where a broad band from approximately −6 eV to −1 eV appears with a peak located around −2.5eV, reaching an occupation of approximately 0.15 states/eV/atom. The Hf *f*-electrons are responsible for a very high peak at low energy values, which is visible in the total EDOS below −11 eV of Hf-rich compositions. These well-bonded states are crucial for the stabilization or the destabilization of the α'' and β phases.

The EDOSs for the orthorhombic structure presents similar features around E_F upon Hf addition, where these are mainly due to the Ti *d*-electron participations with an overall depletion at the highest 25 at.%Hf composition. The different Hf contents result in comparable *d*-EDOSs. For α''-phase 6.25 at.%Hf, electronic states are slightly enhanced around −0.5 eV compared to 25 at.%Hf, while far below E_F the opposite is true. The partial *s*, *p* and *d* states do not present an appreciable difference between the relaxed and unrelaxed system. For α''-Ti-18.75at.%Nb-12.5at.%Hf, the low energy peak stands below −11 eV. This high peak is due to the *f*-Hf contribution, which is only present in the perfect case and is due to the increase of the Hf-Hf distance after the ionic relaxation.

Turning on the β-phase EDOS (Figure S2) the Hf *d*-electrons contributions are responsible for the depletion of the occupied electronic states at E_F, which is critical for the stability of the bcc structure. The *d*-electrons are located between −3.5 eV and E_F being the main responsible for the pseudo-gap at the E_F, while the main *d*-Nb contributions in the EDOS are located around −1eV. The

depletion of states at E_F can be also observed in the Hf and Nb *p*-electrons. The *s*-electrons are shifted towards lower energies as a result of the Hf addition, favoring the β-phase stability. The combination of the depletion of states at E_F and the translation of states to lower energies denotes the stability of the bcc phase as a result of the Hf substitution within the Ti-based matrix. The atomic conformation in which may Hf species are first neighbors is energetically unfavored, however this is difficult to avoid in rich-Hf compositions. The difference between the relaxed and perfect system's EDOS is more pronounced in the β-phase, compared to the orthorhombic, and it is more visible at the states around E_F. In all β-phase compositions, the depletion of electrons in the region around E_F after ionic relaxation leads the system to a more stable structure. This variation is more visible in the relaxed Ti-18.75at.%Nb-25at.%Hf, where the states near E_F are drastically reduced compared to the unrelaxed system.

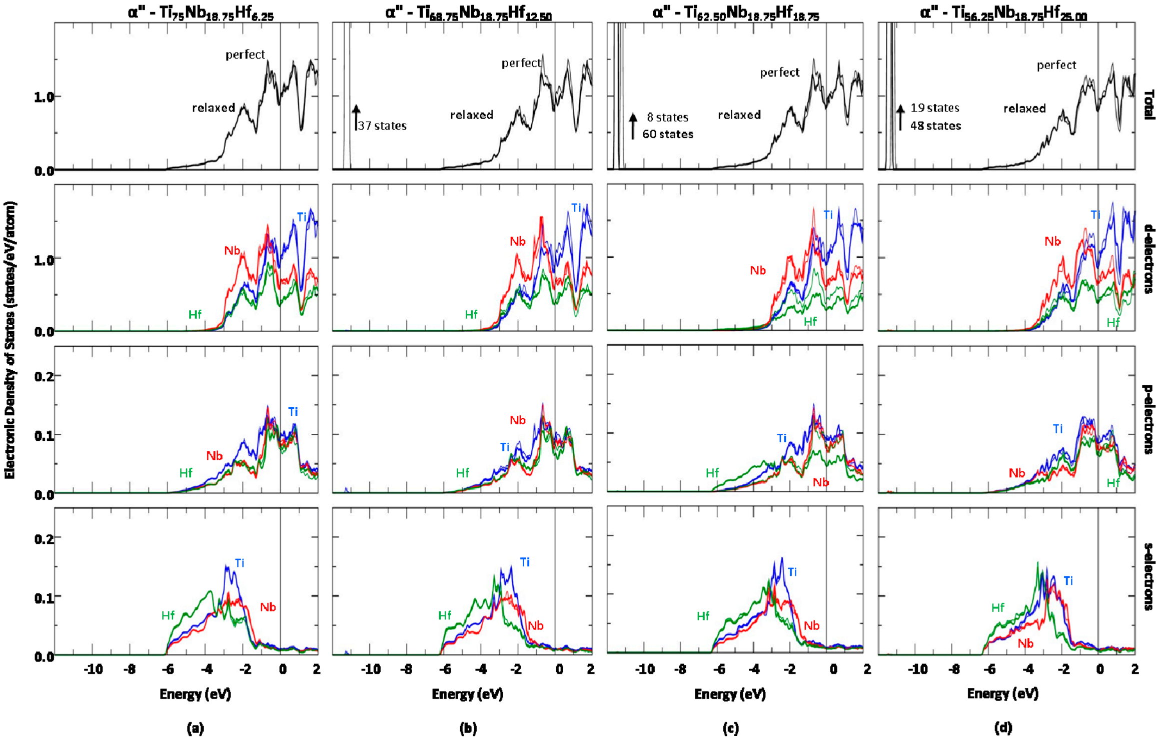

Figure S1. Electronic density of states of α″-phase: (**a**) Ti-18.75at.%Nb-6.25at.%Hf for the total, d, p and s contributions lied up from the first up to fourth row, respectively, (**b**–**d**) stand for the cases of Ti-18.75at.%Nb-12.5at.%Hf, Ti-18.75at.%Nb-18.75at.%Hf and Ti-18.75at.%Nb-25at.%Hf respectively. The contribution due to perfect ideal structure is represented by a thin line and the final structure after ionic relaxation with a thicker trace.

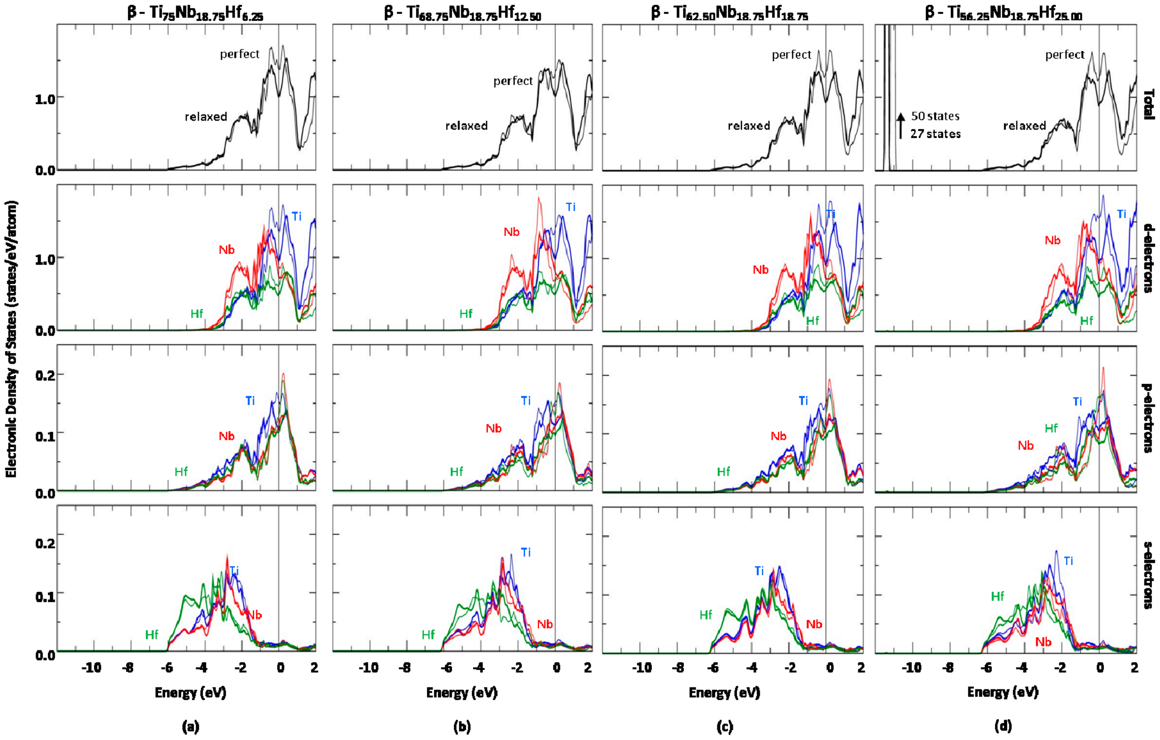

Figure S2. Electronic density of states of β-phase: (**a**) Ti-18.75at.%Nb-6.25at.%Hf for the total, d, p and s contributions lied up from the first up to fourth row, respectively, (**b**–**d**) stand for the cases of Ti-18.75at.%Nb-12.5at.%Hf, Ti-18.75at.%Nb-18.75at.%Hf and Ti-18.75at.%Nb-25at.%Hf respectively. The contribution due to perfect ideal structure is represented by a thin line and the final structure after ionic relaxation with a thicker trace.

© 2020 by the authors. Submitted for possible open access publication under the terms and conditions of the Creative Commons Attribution (CC BY) license (http://creativecommons.org/licenses/by/4.0/).
